# Efficacy of periarticular versus intravenous steroid on postoperative pain and nausea in patients undergoing total knee arthroplasty with local infiltration analgesia: A systematic review and network meta-analysis

**DOI:** 10.1097/MD.0000000000043140

**Published:** 2025-07-18

**Authors:** Sang Gyu Kwak, Jae Bum Kwon, Young Woo Seo, Won-Kee Choi

**Affiliations:** a Department of Medical Statistics, College of Medicine, Daegu Catholic University, Daegu, Korea; b Department of Orthopaedic Surgery, College of Medicine, Daegu Catholic University, Daegu, Korea; c Department of Emergency Medicine, College of Medicine, Daegu Catholic University, Daegu, Korea.

**Keywords:** nausea, pain, steroid, total knee arthroplasty

## Abstract

**Background:**

To compare the effects of postoperative pain relief, nausea relief, and occurrence of adverse effects associated with steroid use between single-dose intravenous steroid (SDIV) and periarticular injection (PAI) of steroid after total knee arthroplasty.

**Methods:**

This systematic review and network meta-analysis was conducted in accordance with PRISMA guidelines. Randomized controlled trials involving patients undergoing primary unilateral total knee arthroplasty with local infiltration analgesia were included. Studies comparing SDIV, PAI, or no steroid use were selected through a comprehensive search of PubMed, Embase, and the Cochrane Library (January 1990–March 2024). Non-English articles, case reports, protocols, and non-randomized controlled trials were excluded. Risk of bias was assessed using the Cochrane Risk of Bias 2.0 tool. A frequentist network meta-analysis was performed to synthesize data on quantitative outcomes (visual analog scale scores, range of motion, flexion angle, opioid consumption) and qualitative outcomes (postoperative nausea and vomiting [PONV], wound complications). Treatment rankings were estimated using surface under the cumulative ranking area values.

**Results:**

Compared to the control group, both SDIV and PAI significantly reduced postoperative visual analog scale scores at rest and during activity up to postoperative day 2. On day 3, SDIV maintained superior pain relief, while PAI effects plateaued. Range of motion and flexion angle were significantly improved in both steroid groups, with PAI showing a slight advantage in early recovery. Opioid consumption was consistently lower in the steroid groups, especially with PAI. Incidence of PONV was lowest in the SDIV group, indicating superior antiemetic effects. No significant differences were observed in wound complication rates across the groups.

**Conclusion:**

In clinical practice, intravenous steroids may be considered for patients at higher risk of PONV, while periarticular steroids may be preferred for enhancing localized pain control in the early postoperative period.

## 1. Introduction

Total knee arthroplasty is one of the orthopedic surgeries with high postoperative patient satisfaction.^[1]^ However, severe postoperative pain and frequent nausea and vomiting diminish patient satisfaction significantly.^[2,3]^ Recently, steroids have been widely used for their anti-inflammatory effects to reduce postoperative pain and nausea.^[4,5]^ Numerous studies have demonstrated the efficacy of various glucocorticoid administration routes and regimens in postoperative pain management and nausea relief.^[6,7]^ However, there remains disagreement regarding the most effective administration route.^[8]^ Therefore, the authors aim to conduct a network meta-analysis to compare the effects of postoperative pain relief, nausea relief, and occurrence of adverse effects associated with steroid use between the commonly used systemic route, intravenous route, and local route, periarticular route. The authors aim to investigate the following points.

1. For primary unilateral total knee arthroplasty, we aim to compare and analyze the effectiveness of pain control postoperatively among 3 groups: single-dose intravenous steroid (SDIV), periarticular steroid injection (PAI) with local infiltration analgesia (LIA),^[9]^ and a group without steroid use. Additionally, we seek to compare the amount of opioid used for pain control among these groups.
2. For primary unilateral total knee arthroplasty, we aim to compare and analyze the postoperative range of motion of knee among 3 groups: SDIV, PAI with LIA, and a group without steroid use.
3. For primary unilateral total knee arthroplasty, we aim to compare and analyze the efficacy of postoperative nausea among 3 groups: SDIV, PAI with LIA, and a group without steroid use.

## 2. Materials and methods

### 2.1. Registration and reporting guidelines

Since this network meta-analysis is not a clinical study involving human subjects, a protocol for IRB approval and ethics approval was not needed. Instead, the authors wrote protocol to carry out meta-analysis and registered the protocol on the international prospective register of systematic reviews site (https://www.crd.york.ac.uk/PROSPERO/) at June 11, 2024 (ID: CRD42024556961). Also, transparent reporting of systemic review and meta-analysis (PRISMA Checklist: http://prisma-statement.org/) was completed.

### 2.2. Search strategy

The PICO (population, intervention, comparison, and outcome) of this study are as follows.

(1) Population: patients who underwent primary unilateral total knee arthroplasty with LIA.
(2) Intervention: steroid use (SDIV or PAI).
(3) Comparison: no steroid use.
(4) Outcome: changes visual analog scale (VAS) at rest before surgery and after surgery, changes VAS during activity before surgery and after surgery, range of motion (ROM) of knee, postoperative nausea and vomiting (PONV), use of postoperative opioid consumption, and wound complication.

Articles published between January 1, 1990, and March 30, 2024 were searched in PubMed, Cochrane, and Embase using the following key phrases (Table 1).

**Table 1:** Search keywords by database.

A. PubMed	
Search	Query
#1	“Total knee arthroplasty”[Ti/Ab] OR “Total knee replacement”[Ti/Ab]
#2	“dexamethasone”[Ti/Ab] OR “steroid”[Ti/Ab] OR “corticosteroid”[Ti/Ab] OR “corticosteroids”[Ti/Ab] OR “triamcinolone”[Ti/Ab]
#4	#1 AND #2
Articles were searched in PubMed using the following key phrases.	
B. Cochrane Library	
Search	Query
#1	MeSH descriptor: [Arthroplasty, Replacement, Knee] explode all trees
#2	(“total knee arthroplasty”):ti,ab OR (“total knee replacement”):ti,ab
#3	MeSH descriptor: [Adrenal Cortex Hormones] explode all trees
#4	(“dexamethasone”):ti,ab OR (“steroid”):ti,ab OR (“corticosteroid”):ti,ab OR (“corticosteroids”):ti,ab OR (“triamcinolone”):ti,ab
#5	#2 OR #1
#6	#4 OR #3
#7	#5 AND #6
	C. Embase
Search	Query
1	“knee arthroplasty”/exp OR “total knee arthroplasty”:ti,ab OR “total knee replacement”:ti,ab
2	“adrenal cortex hormone”/exp OR dexamethasone:ti,ab OR steroid:ti,ab OR corticosteroid:ti,ab OR corticosteroids:ti,ab OR triamcinolone:ti,ab
3	1 AND 2

Inclusion and exclusion criteria

The following studies were included in this study:

(1) Studies involving patients undergoing primary unilateral total knee arthroplasty (TKA) with LIA.
(2) Studies comparing steroid of SDIV versus no steroid, steroid of PAI versus no steroid, or steroid SDIV versus steroid of PAI.
(3) Intervention group using steroids of intermediate acting or long acting.^[10]^
(4) Studies where the dosage of dexamethasone used was 20 mg or less.^[11]^
(5) Studies implementing a single-dose intravenous steroid regimen (SDIV).
(6) Studied of randomized controlled trials (RCTs).

The exclusion criteria were as follows:

(1) Review articles, case reports, protocols, and conference presentations.
(2) Studies published in languages other than English.

### 2.3. Data extraction

Data for network meta-analysis (NMA) were independently investigated by 2 researchers (WKC and SGK). Duplicate studies were excluded, and studies that met the eligibility criteria were selected. Studies were evaluated for eligibility by reviewing the title and abstract. After reading the full text, studies were finally selected for inclusion in the NMA, and discrepancies were resolved through discussion. Research characteristics, study design, number of patients (PAI, SDIV, and control groups), dose of steroid, type of steroid injection, outcome measurements, time, and number of measurements were investigated.

Outcome variables were the quantitative variables of VAS at rest (0–10), pain VAS during activity (0–10), range of motion (ROM), flexion angle (°), opioid (morphine, tramadol, etc) consumption, and the qualitative variables of PONV, wound complication (Y/N). In the papers, when the range of VAS was expressed as 0 to 100, it was divided by 10 and unified as 0 to 10. When the median (m), minimum value (a), and maximum value (b) were presented for a quantitative variable, the mean and standard deviation (SD) values were calculated using the following formula.^[12]^


$$\begin{array}{l} Mean=\frac{a+2m+b}{4}\;\;\;and\;\;\;SD=\sqrt{\frac{1}{12}\left(\frac{{\left(a-2m+b\right)}^{2}}{4}+{\left(b-a\right)}^{2}\right)}. \end{array}$$

when the mean and the upper bound and lower bound values of 95% confidence interval (CI) were presented, the standard deviation (SD) value was calculated using the following formula. Here, $z_{\alpha /2}$ is the value of the point corresponding to α/2 on the standard normal distribution.


$$\begin{array}{l} SD=\frac{\left(Upper\;\;\;bound-mean\right)}{z_{\alpha /2}}\times \sqrt{n}. \end{array}$$

when only the mean values of each group and significance probability (*P*-value) were presented without the standard deviation value, the standard deviation value was calculated using the following formula. Here, ${\;\Phi \;}^{-1}()$ is the inverse function of the cumulative distribution function in the t-distribution.


$$\begin{array}{l} SD=\frac{\left(\sqrt{n}\times mean\;difference\right)}{{\;\Phi \;}^{-1}\left(1-\frac{p-value}{2}\right)}. \end{array}$$

when the range (*r*) using the minimum and maximum values was presented, the mean and standard deviation (SD) values were calculated using the following formula.^[13]^ Here f is the tabulated conversion factor.


$$\begin{array}{l} SD=f\times r. \end{array}$$

when statistical values such as mean and SD were not presented in a table but only as a graph, the exact numerical value was extracted from the graph using the Engauge Digitizer software (version 12.1, Webpage: https://engauge-digitizer.software.informer.com/4.1/). Additionally, when the values presented in the paper were insufficient, we contacted the authors of the paper and received the necessary values for use.

We collected the values of the VAS at rest and VAS during activity presented in the paper according to the evaluation time points. Then, the changes before surgery and 24 hours after surgery, before surgery and 48 hours after surgery, and before surgery and 72 hours after surgery were calculated. Since this corresponds to the amount of change in all groups, the mean value and standard deviation for changes before surgery and after surgery were calculated using the following formulas and the correlation was derived from different study which presented the standard deviation for change.


$$\begin{array}{l} Mean_{change}=Mean_{before\;surgery}-Mean_{after\;surgery}. \end{array}$$

$$\begin{array}{lll} & SD_{change}=\; & \sqrt{SD_{before\;surgery}^{2}+SD_{after\;surgery}^{2}-2\times Correlation\times SD_{before\;surgery}\times SD_{after\;surgery}}. \end{array}$$

### 2.4. Quality assessment

The methodological quality was evaluated using revised Cochrane risk of bias tool (ROB 2.0). The potential sources of bias are listed in the followings; bias arising from the randomization process (D1), bias due to deviations from intended intervention (D2), bias due to missing outcome data (D3), bias in measurement of the outcome (D4), and bias in selection of the reported result (D5). The judgement of bias was described as one of the followings: “low risk of bias,” “some concerns,” “high risk of bias,” or “no information.”^[14]^ These evaluations were conducted by 2 independent reviewers (SGK and WGC), and all discrepancies were resolved through discussions between them.

### 2.5. Data synthesis and analysis

We constructed NMA models using a frequentist framework. The statistical approach is derived from graph theoretical methods developed for electrical networks. In this meta-analysis, we applied either a fixed effects model or a random effects model based on the degree of heterogeneity. Specifically, a fixed effects model was used when the studies were sufficiently homogeneous (i.e., low I² value and no statistically significant heterogeneity), whereas a random effects model was adopted when substantial heterogeneity was present. To perform a network meta-analysis, we need a connected network to make comparisons among PAI, SDIV, and control group. We analyzed 5 quantitative variables and 2 qualitative variables. Pain VAS in rest, VAS during activity, ROM, flexion, opioid consumption was analyzed as quantitative variables. PONV and wound complication were analyzed as qualitative variables. In the case of pain VAS in rest and VAS during activity, the amount of change before and after surgery was calculated. For this purpose, the difference VAS between before and 24 hours after surgery, the difference between before and 48 hours after surgery, and the difference between before and 72 hours after surgery were calculated. Among the VAS presented in the paper, values measured at 3 hours, 6 hours, 9 hours, 12 hours, 15 hours, 18 hours, and 21 hours after surgery were included before surgery and 24 hours after surgery. Among the VAS presented in the paper, values measured at 30 hours, 33 hours, 36 hours, 39 hours, 42 hours, and 45 hours after surgery were included before surgery and 48 hours after surgery. Therefore, we analyzed eleven quantitative variables and 2 qualitative variables, finally. The I^2^ statistic and Cochran Q test were used to determine the heterogeneity of direct comparisons, and significant heterogeneity was assumed in the case of I^2^ values > 50% and *P*-values < .05. Forest plots of pairwise comparison were presented and the outcomes are presented as standard mean differences (SMDs) and 95% CI for the quantitative variables and odds ratio (OR) and 95% CI for the qualitative variables. Network plots were presented in all cases and the thickness of the edge indicates the amount of data. Forest plots of network comparisons were presented as number of studies, direct evidence, and direct estimate. Publication bias was examined using Egger regression test and by inspecting the distribution pattern of the effect size on the funnel plot. The probability ranking metrics were used to reflect the clinically important relative differences in the outcomes, which were shown on the ranking probability curves and surface under the cumulative ranking area (SUCRA). The SUCRA value ranged between 0 and 1, and treatments with a higher SUCRA value suggested superior ranking. Therefore, higher SUCRA value suggested better effectiveness for the changes in VAS at rest (preop-24 hour, preop-48 hour, preop-72 hour), changes in VAS during activity (preop-24 hour, preop-48 hour, preop-72 hour), ROM and flexion angle. Lower SUCRA value better suggested better effectiveness for opioid consumption, PONV, and wound complication. It was presented as the percentage of the mean rank of each treatment in relation to the presumed best intervention. All analyses were performed using the “net meta” package in R software (R version 4.3.2) and *P*-values were <.05 were considered statistically significant.

## 3. Results

### 3.1. Study selection and characteristics

**Figure 1.**
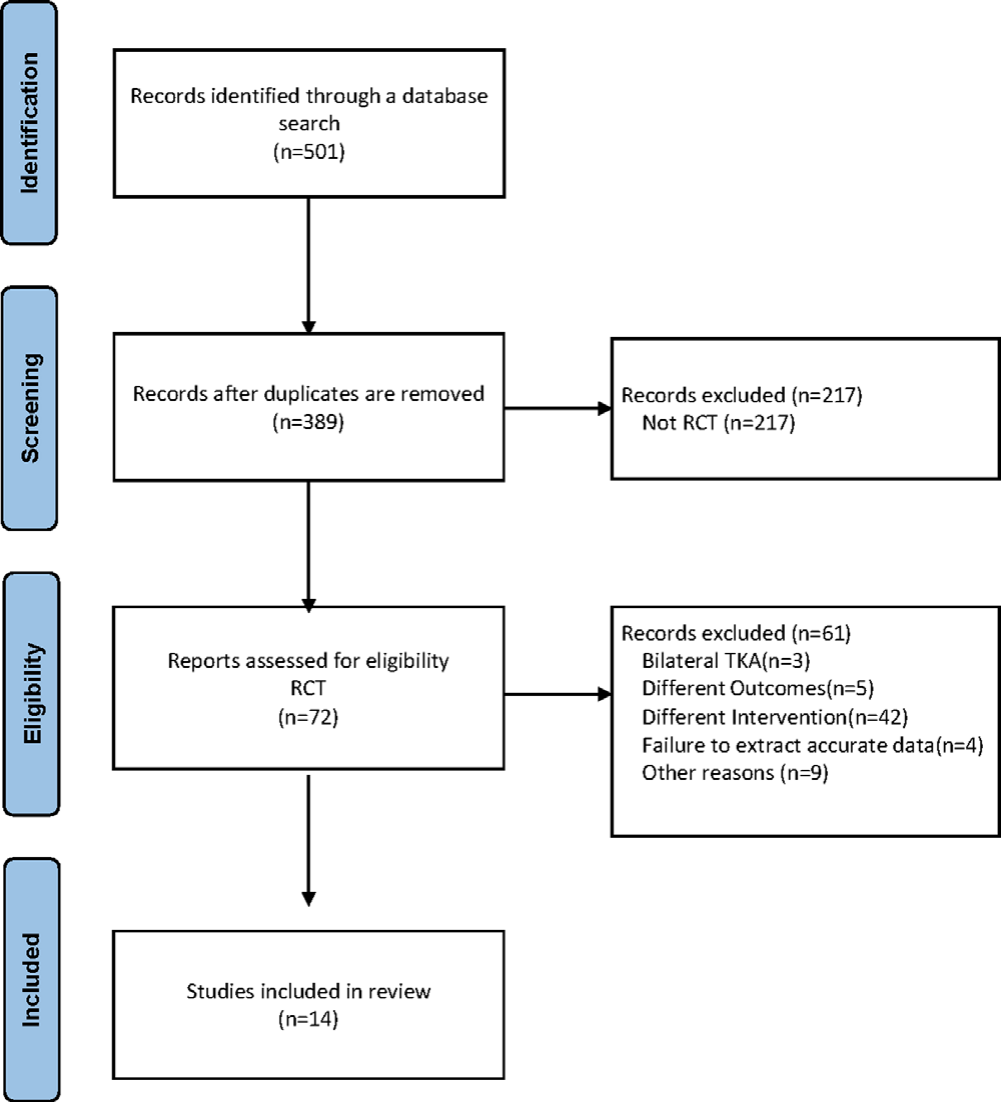
Flow diagram of the study selection process.

In total, 501 articles were identified as potentially relevant in the primary literature search (Fig. 1). After reviewing the titles and abstracts and assessing their eligibility based on the full text, 14 studies were included in the network meta-analysis^[4,7,15–26]^ (Table 2).

**Table 2:** Characteristics of included RCTs.

Reference	Comparison	Number of patients	Types of steroids/dose (mg)	Dose conversion to Dexamethasone (mg)	Outcome measurements	Measurement time of pain score	Measurement time of knee ROM
Christensen^[20]^	PAI vs no steroid	39/37	Methylprednisolone/40	7.5	Pain score Knee ROM Opioid consumption	Pre OP POD 1	
Chia^[22]^	PAI vs no steroid	42/43 42/43	Triamcinolone/40 Triamcinolone/80	7.5 15	Knee ROM		Pre OP 2 wk
Ikeuchi^[21]^	PAI vs no steroid	20/20	Dexamethasone/6.6	6.6	Pain score Opioid consumption PONV	Pre OP POD 1 POD 3	
Tsukada^[23]^	PAI vs no steroid	40/37	Methylprednisolone/40	7.5	Pain score Knee ROM	Pre OP POD 1 POD 2 POD 3	Pre OP POD 1 1 wk 2 wk
El-Boghdadly^[19]^	PAI vs no steroid	68/72	Dexamethasone/8	8	Pain score Knee ROM PONV Wound complication Opioid consumption	Pre OP POD 0 POD 1 POD 2 POD 3	Pre OP POD 0 POD 1 POD 2 POD 3
Wang^[18]^	PAI vs no steroid	52/50	Dexamethasone/10	10	Pain score Knee ROM PONV Wound complication Opioid consumption	Pre OP post 2 h post 6 h post 12 h post 24 h post 48 h	Pre OP POD 1 POD 2 POD 3
Saini^[24]^	PAI vs SDIV	60/60	PAI: Dexamethasone/8 SDIV: Dexamethasone/8	8	Pain score Knee ROM PONV Wound complication	Pre OP post 24 h post 48 h	Pre OP POD 2
Chan^[25]^	PAI vs no steroid	44/45	PAI: Triamcinolone/40	7.5	Pain score Knee ROM Opioid consumption	Pre OP POD 1 POD 2 POD 3	POD 1 POD 2 POD 3
	SDIV vs no steroid	44/45	SDIV: Dexamethasone/16	16			
	PAI vs SDIV	44/44	PAI: Triamcinolone/40 SDIV: Dexamethasone/16	7.5 16			
Li^[26]^	PAI vs SDIV	45/45	PAI: dexamethasone/10 SDIV: dexamethasone/10	10 10	Pain score Knee ROM Opioid consumption Wound complication	Pre OP Post 2 h Post 6 h Post 12 h Post 24 h Post 48 h	POD 1 POD 2
Xu^[17]^	SDIV vs no steroid	60/61	Dexamethasone/20	20	Pain score Knee ROM PONV Wound complication	Pre OP POD 1 POD 2 POD 3	POD 3 POD 15 POD 90
Tammachote^[7]^	SDIV vs no steroid	50/50	Dexamethasone/0.15 mg per kg	maximum dose 12	Pain score PONV Opioid consumption	Pre OP Every 3 hours for post 48 hours	
Chan^[16]^	SDIV vs no steroid	46/45	Dexamethasone/8 Dexamethasone/16	8 16	Pain score Knee ROM Opioid consumption Wound complication	Pre OP POD 1 POD 2 POD 3	Pre OP POD 1 POD 2 POD 3
Kim^[4]^	SDIV vs no steroid	45/44	Dexamethasone/10	10	Pain score Wound complication	Pre OP POD 1 POD 2 POD 3 POD 5 POD 7	
Lei^[15]^	SDIV vs no steroid	62/63	Dexamethasone/20	20	Pain score Knee ROM Wound complication	Pre OP POD 1 POD 2 POD 3	Pre OP POD 3

OP = operation, PAI = periarticular injection, POD = post-operative day, PONV = post-operative nausea and vomiting, RCTs = randomized controlled trials, ROM = range of motion, SDIV = single-dose intravenous injection.

Among 14 studies included, 6 compared PAI and control, 5 compared SDIV and control, 2 studies compared PAI and SDIV, and 1 study compared all 3 groups. This network meta-analysis included 1389 participants, of which 410, 412, and 567 participants received PAI, SDIV, and control, respectively.

The steroids used in the PAI group included Methylprednisolone (40 mg), Triamcinolone (40 mg and 80 mg), and Dexamethasone (6.6 mg, 8 mg, and 10 mg). In the SDIV group, all studies used Dexamethasone, with doses ranging from 8 mg to 20 mg.

Outcome measures included VAS at rest (12 studies), VAS during activity (9 studies), ROM (6 studies), flexion angle (7 studies), opioid consumption (9 studies), PONV (7 studies), and wound complications (7 studies). Comparisons were made across different doses, time points, and groups.

### 3.2. Results of the network meta-analysis

The values of I^2^ and *P*-values in Cochran Q test were performed. When the *I*^2^ value was more than 50% and the *P*-value was <.05, it was analyzed as a random-effect model. In the opposite case, it was analyzed as a common-effect model. The random-effect model was used for changes in VAS at rest, VAS during activity, ROM, and flexion angle. The results are follows: VAS at rest changes between before surgery and within 24 hours after surgery (preop-24 hour), I^2^ = 61.9% and *P*-value < .001; VAS at rest (preop-48 hour), I^2^ = 57.0% and *P*-value = .001; VAS at rest (preop-72 hour), I^2^ = 85.9% and *P*-value < .001; VAS during activity (preop-24 hour), I^2^ = 69.2% and *P*-value < .001; VAS during activity (preop-48 hour), I^2^ = 43.2% and *P*-value = .027; VAS during activity (preop-72 hour), I^2^ = 70.7% and *P*-value = .001; ROM, I^2^ = 61.8% and *P*-value = .005; flexion angle, I^2^ = 48.6% and *P*-value = .009. The common-effect model was used for opioid consumption, PONV, and wound complication. The results are follows: opioid consumption, I^2^ = 0.0% and *P*-value = .673; PONV, I^2^ = 0.0% and *P*-value = .491; wound complication, I^2^ = 0.0% and *P*-value = .967.

**Figure 2.**
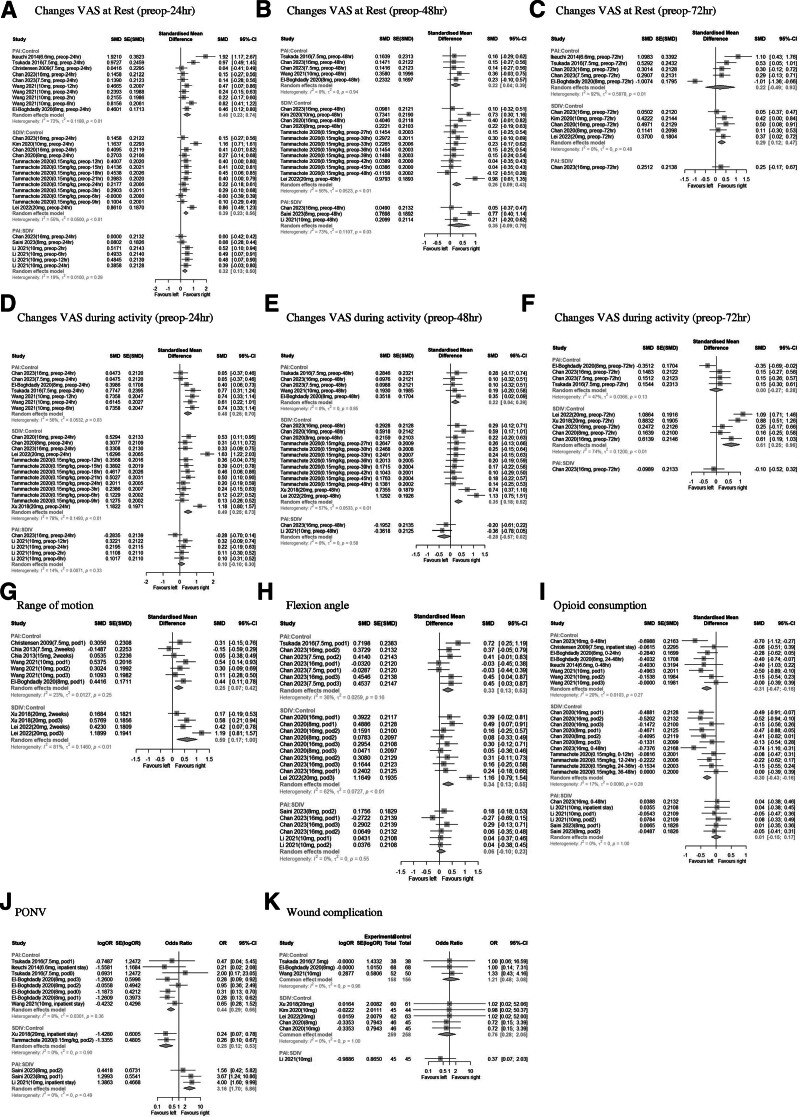
Forest plots of pairwise comparison. The steroid dose and duration or time point of steroids are presented in parentheses, and the steroid dose is presented by converting it to dexamethasone. CI = confidence interval, OR = odds ratio, PAI = periarticular injection, PONV = post-operative nausea and vomiting, SDIV = single-dose intravenous injection, SMD = standard mean difference, VAS = visual analog scale.

Forest plots of pairwise comparisons indicated significant differences in VAS at rest and during activity between PAI, SDIV, and control at certain time points. ROM and flexion angle also showed significant differences between treatments, while opioid consumption showed no significant difference between PAI and SDIV. PONV had significant differences, whereas wound complications showed no difference (Fig. 2).

**Figure 3.**
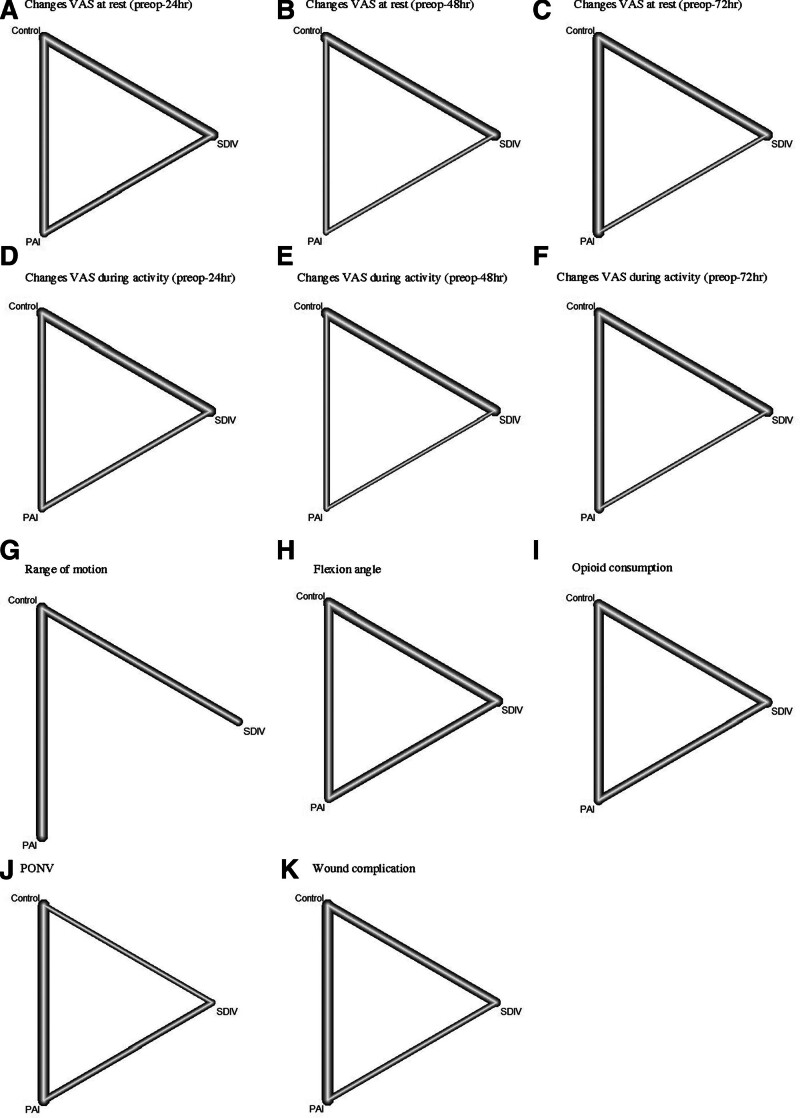
Network plots of PAI, SDIV, and control group. PAI = periarticular injection, PONV = post-operative nausea and vomiting, SDIV = single-dose intravenous injection, VAS = visual analog scale.

Network analysis confirmed pairwise results with additional findings: SDIV showed larger differences compared to control in VAS and flexion angle, while PAI performed better for opioid consumption and ROM. PONV was lower for PAI and SDIV compared to control. Wound complications had no significant (Fig. 3).

**Figure 4.**
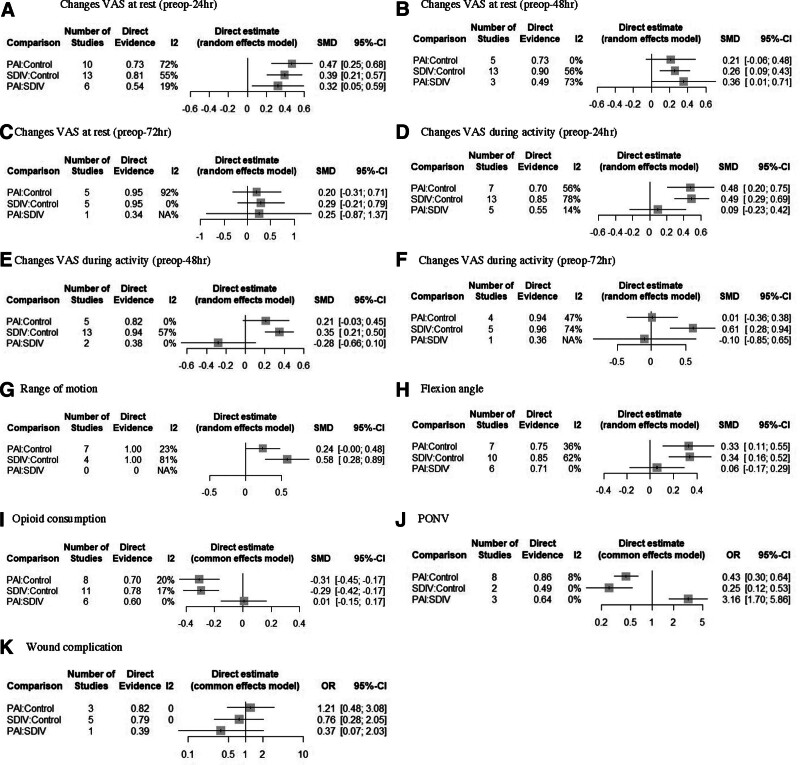
Forest plots of network comparisons. CI = confidence interval, NA = not applicable, OR = odds ratio, PAI = periarticular injection, PONV = post-operative nausea and vomiting, SDIV = single-dose intravenous injection, SMD = standard mean difference, VAS = visual analog scale.

Regarding VAS during activity changes between before surgery and within 24 hours after surgery (preop-24 hour), PAI and SDVI have a significantly larger difference compared to control (PAI vs control: SMD = 0.48, 95% CI = 0.20–0.75; SDIV vs control: SMD = 0.49, 95% CI = 0.29–0.69) but there is no difference between PAI and SDVI (SMD = 0.09, 95% CI = -0.23 to 0.42; Fig. 4D). For VAS during activity (preop-48 hour), SDIV has a significantly larger difference compared to control (SMD = 0.35, 95% CI = 0.21–0.50) but there is no difference between PAI and control (SMD = 0.21, 95% CI = -0.03 to 0.45), PAI and SDIV (SMD = -0.28, 95% CI = -0.66 to 0.10; Fig. 4E). For VAS during activity (preop-72 hour), SDIV has a significantly larger difference compared to control (SMD = 0.61, 95% CI = 0.28–0.94) but there is no difference between PAI and control (SMD = 0.01, 95% CI = -0.36 to 0.38), PAI and SDIV (SMD = -0.10, 95% CI = -0.85 to 0.65; Fig. 4F). Regarding flexion angle, PAI and SDVI have a significantly larger difference compared to control (PAI vs control: SMD = 0.33, 95% CI = 0.11–0.55; SDIV vs control: SMD = 0.34, 95% CI = 0.16–0.52) but there is no difference between PAI and SDVI (SMD = 0.06, 95% CI = -0.17 to 0.29; Fig. 4H). Regarding opioid consumption, PAI and SDVI have a significantly smaller difference compared to control (PAI vs control: SMD = -0.31, 95% CI = -0.45 to -0.17; SDIV vs control: SMD = -0.29, 95% CI = -0.42 to -0.17) but there is no difference between PAI and SDVI (SMD = 0.01, 95% CI = -0.15 to 0.17; Fig. 4I). Regarding PONV, PAI, and SDVI have a significantly smaller compared to control (PAI vs control: OR = 0.43, 95% CI = 0.30–0.64; SDIV vs control: OR = 0.25, 95% CI = 0.12–0.53) and PAI has a significantly larger compared to SDIV (OR = 3.16, 95% CI = 1.70–5.86; Fig. 4J). Regarding wound complication, there are no significant differences for all pairwise comparisons (Fig. 4K).

**Figure 5.**
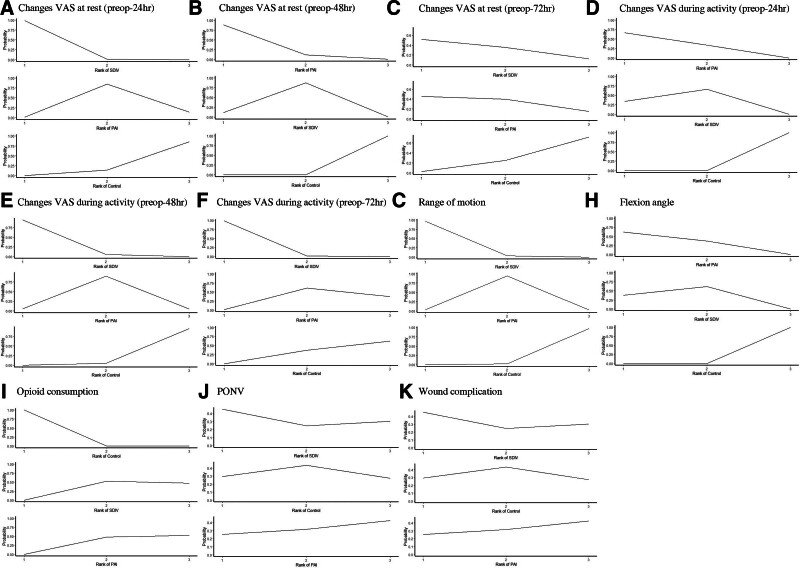
Ranking probabilities for PAI, SDIV, and control. PAI = periarticular injection, PONV = post-operative nausea and vomiting, SDIV = single-dose intravenous injection, VAS = visual analog scale.

The rank probability results and SUCRA values are shown in Figure 5 and Table 3. Rank probability analysis showed SDIV was the best treatment for VAS at rest (preop-72 hour) and ROM, while PAI was best for VAS at rest (preop-24 hour and preop-48 hour), VAS during activity (preop-24 hour), and flexion angle. Control had the highest likelihood of being the worst for PONV and wound complications.

**Table 3:** Surface under the cumulative ranking (SUCRA) of the changes in pain VAS at rest (preop-24 hour, preop-48 hour, and preop-72 hour), changes in VAS during activity (preop-24 hour, preop-48 hour, preop-72 hour), ROM, flexion angle, opioid consumption, PONV, and wound complication.

Variable	Rank	Group	SUCRA
Changes in pain VAS at rest (preop-24 h)	1	PAI	0.992
	2	SDIV	0.508
	3	Control	0.000
Changes in pain VAS at rest (preop-48 h)	1	PAI	0.931
	2	SDIV	0.564
	3	Control	0.005
Changes in pain VAS at rest (preop-72 h)	1	SDIV	0.701
	2	PAI	0.638
	3	Control	0.154
Changes in VAS during activity (preop-24 h)	1	PAI	0.848
	2	SDIV	0.652
	3	Control	0.000
Changes in VAS during activity (preop-48 h)	1	SDIV	0.969
	2	PAI	0.507
	3	Control	0.024
Changes in VAS during activity (preop-72 h)	1	SDIV	0.994
	2	PAI	0.344
	3	Control	0.163
ROM	1	SDIV	0.986
	2	PAI	0.501
	3	Control	0.013
Opioid consumption	1	Control	1.000
	2	SDIV	0.266
	3	PAI	0.234
Flexion angle	1	PAI	0.820
	2	SDIV	0.681
	3	Control	0.000
PONV	1	Control	1.000
	2	PAI	0.501
	3	SDIV	0.000
Wound complication	1	SDIV	0.561
	2	Control	0.529
	3	PAI	0.410

PAI = periarticular injection, PONV = postoperative nausea and vomiting, ROM = range of motion, SDIV = single-dose intravenous steroid, SUCRA = surface under the cumulative ranking area, VAS = visual analog scale.

### 3.3. Publication bias

**Figure 6.**
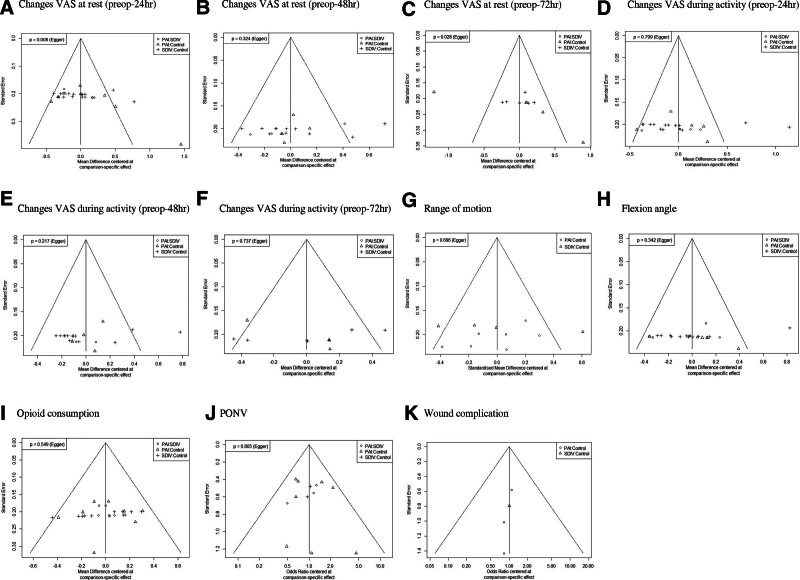
Graphical funnel plots of the included studies. PAI = periarticular injection, PONV = post-operative nausea and vomiting, SDIV = single-dose intravenous injection, VAS = visual analog scale.

On the basis of a few distinct methods, 2 of the authors (WKC and SGK) individually assessed the risk of bias. The risk of publication bias was determined using a funnel plot and the Egger test. A funnel plot was produced to investigate the risk of publication bias. Performing a visual inspection suggested some funnel plot asymmetry (Fig. 6). In addition, the results of Egger test were not statistically significant (*P*-value > .05) for all variables except changes VAS at rest and VAS during activity.

### 3.4. Qualitative synthesis

Before conducting the quantitative meta-analysis, we performed a qualitative synthesis to assess the clinical and methodological characteristics of the included studies and to identify potential sources of heterogeneity or bias.

#### 3.4.1. Clinical and methodological characteristics (Standard 4.2.1)

This systematic review includes a total of 14 RCTs (Table 2), each evaluating the effectiveness of steroid medications for pain control and functional recovery following total knee arthroplasty. The studies were designed with interventions based on the route of steroid administration, including PAI, SDIV, or a comparison group (no steroid). Most studies assessed outcomes such as pain scores, knee ROM, opioid consumption, nausea/vomiting (PONV), and wound complications at various time points before and after the surgery. The steroids used included Dexamethasone, Methylprednisolone, and Triamcinolone, with doses converted to Dexamethasone equivalents ranging from 6.6 mg to 20 mg. The sample sizes ranged from 40 to 135 participants (a total of 2324 participants), with most studies employing a 1:1 ratio for experimental and control groups. The evaluation times varied between studies, with pain measured from immediately after surgery up to 3 days or 1 week, while ROM was followed up for up to 12 weeks in some studies.

#### 3.4.2. Strengths and limitations of individual studies (Standard 4.2.2)

Most studies included in this review utilized random assignment and clearly defined intervention groups (e.g., PAI vs no steroid, SDIV vs no steroid, PAI vs SDIV) to assess the effects of the intervention. However, several limitations were common across studies:

Limited sample size: some studies (e.g., Ikeuchi 2014, Christensen 2009) had fewer than 80 participants, which limited statistical power. Heterogeneity in Evaluation Timepoints: The timing of pain score and ROM measurements varied between studies, making direct comparisons difficult. Diversity in steroid type and dose: the use of different steroids (Methylprednisolone, Triamcinolone, Dexamethasone) and varying doses made it challenging to estimate a consistent effect. Inconsistency in reporting adverse events: not all studies consistently reported wound complications or PONV.

#### 3.4.3. Potential bias from design or execution (Standard 4.2.3)

Although most studies were RCTs, some studies exhibited potential bias, including:

Lack of blinding: some studies did not mention whether blinding was used for patients or assessors, introducing the possibility of observer bias. Incomplete follow-up: studies with short follow-up periods or inadequate reporting on loss to follow-up weakened the reliability of long-term outcomes. Lack of standardization in intervention: variations in the composition and administration methods of PAI across studies could undermine internal validity in effect comparisons.

#### 3.4.4. Relationships between study characteristics and outcomes (Standard 4.2.4)

The route and dose of steroid administration were significant factors influencing pain reduction and ROM improvement. For instance, studies administering 10 to 20 mg of Dexamethasone via SDIV (Lei^[15]^, Xu^[17]^, Kim^[4]^) frequently reported greater prevention of PONV and wound complications compared to PAI alone. Conversely, PAI tended to show more favorable outcomes in terms of ROM improvement and reduced opioid use (Chia^[22]^, Chan^[25]^). Moreover, studies using high-dose (≥16 mg) Dexamethasone indicated more pronounced pain control effects, though adverse events were reported less frequently.

#### 3.4.5. Relevance to population and outcomes (Standard 4.2.5)

All studies focused on adults undergoing total knee arthroplasty, and most were based on typical clinical scenarios (primary arthroplasty, single-dose steroid administration, standard surgical procedures), enhancing the clinical applicability of the results. Additionally, the primary outcomes of most studies (pain scores (primarily VAS), knee ROM, opioid use, and adverse events (PONV, wound complications)) were clinically meaningful, further supporting the relevance of the findings to patient care.

### 3.5. Assessment of the study quality

**Figure 7.**
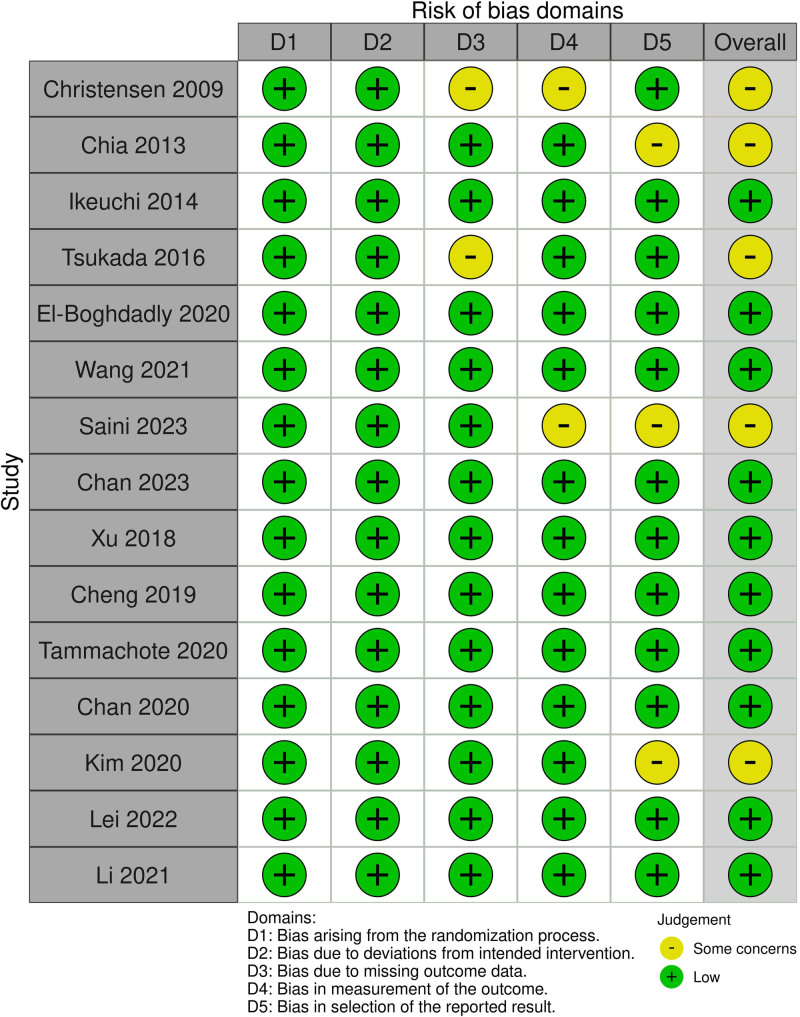
Graphical plot for assessment of the study quality.

The included studies were evaluated for 5 domains (D1–D5) using ROB 2.0 and robvis (risk of bias visualization tool: https://www.riskofbias.info/welcome/robvis-visualization-tool) (Fig. 7). Since all papers are RCTs, 2 out of 5 categories “bias arising from the randomization process (D1)” and “bias due to deviations from intended intervention (D2)” were evaluated as low risk of bias. In 2 studies, the category “bias due to missing outcome data (D3)” was evaluated as some concern because there was no technology for how to deal with missing values even though there were missing values at the time of follow up in the results. In 2 studies, the “bias in outcome measurement (D4)” category was evaluated as having some concern because there was no precise description of whether pain scores were at rest or during activity. In 3 studies, the “bias in selection of the reported result (D5)” category was evaluated as having some concern. The first study presented cumulative RVAS and AVAS, resulting in loss of information, and the second study only presented the difference in VAS between 2 groups and did not present VAS in each group. The third study present mean without standard deviation and did not accurately present the *P*-value, which is the result of comparison between the 2 groups. Therefore, the overall evaluation result among the 14 papers is that 9 papers were evaluated as “low risk of bias,” and 5 papers were evaluated as “some concerns.”

## 4. Discussion

This study provides novel comparative insights into the relative effectiveness of periarticular versus intravenous steroid administration following TKA. While prior studies have investigated each method separately, our network meta-analysis is the first to integrate pain, nausea, opioid usage, functional recovery, and complications into a single comparative framework.

The objective of this study was to investigate the efficacy of steroid use in relieving pain and nausea following TKA, and to assess whether the effectiveness varies depending on the mode of steroid administration (systemic intravenous [SDIV] vs PAI) through network meta-analysis. The primary findings are summarized as follows.

Pain intensity was evaluated using VAS scores on postoperative days 1, 2, and 3, under resting and activity conditions. To adjust for interindividual variability in baseline pain perception, the change in VAS scores from preoperative levels was calculated for comparison across groups. Up to postoperative day 2, both the PAI and SDIV groups demonstrated significantly greater reductions in VAS scores (rest and activity) compared to the no steroid group, indicating enhanced analgesic effects. On postoperative day 3, the SDIV group continued to show a significant reduction in VAS scores compared to the control, whereas the PAI group did not. When directly comparing the 2 steroid administration routes, PAI was associated with a significantly greater reduction in resting VAS on postoperative day 1. No other time points or conditions demonstrated statistically significant differences between the PAI and SDIV groups. Notably, none of the intergroup differences exceeded the minimal clinically important difference of 0.9 points for VAS following TKA, suggesting limited clinical relevance despite statistical significance.^[27]^

Knee functional recovery was evaluated in terms of overall ROM and flexion angle. Both steroid groups (PAI and SDIV) exhibited improved postoperative ROM and flexion compared to the control group. However, no significant differences were observed between the PAI and SDIV groups in terms of joint angle recovery, implying comparable functional outcomes irrespective of administration route.

Regarding PONV, both PAI and SDIV groups experienced fewer PONV episodes relative to the non-steroid group. Moreover, the SDIV group demonstrated a lower incidence of PONV than the PAI group, indicating superior antiemetic efficacy of systemic steroid administration.

In the context of opioid consumption, both steroid-treated groups showed reduced opioid requirements for postoperative pain management compared to the control. However, opioid usage did not differ significantly between the SDIV and PAI groups. No significant differences in wound-related complications were identified across the groups, suggesting that steroid administration, regardless of the route, did not increase the risk of adverse events.

This study implemented a network meta-analysis to simultaneously compare 3 groups: PAI, SDIV, and no steroid use. It is noteworthy that most studies involving PAI employed LIA in conjunction with steroids, as LIA is a standard modality for postoperative pain relief. To ensure valid comparisons, we included only studies that employed LIA across all groups, thereby minimizing the confounding effect of analgesic technique.

Several factors may have influenced the outcomes of this analysis. Heterogeneity in steroid types, dosages, and durations of action could affect analgesic efficacy. This study only included intermediate- and long-acting steroids, with dosages equivalent to ≤ 20 mg of dexamethasone to reduce variability.^[11]^ Among the 8 studies in the PAI group, 4 utilized intermediate-acting steroids such as methylprednisolone and triamcinolone (duration: 24–36 hours),^[28]^ while the remaining 4 used long-acting prednisolone. In contrast, all studies in the SDIV group employed long-acting prednisolone. These pharmacokinetic differences may have contributed to observed outcome variations.

From a pharmacophysiological standpoint, PAI delivers steroids directly into the surgical site, achieving high local concentrations while minimizing systemic exposure. This local delivery modulates inflammation by suppressing pro-inflammatory cytokines and reducing neutrophil migration in periarticular tissues. The sustained local presence of steroids also supports extended analgesia in the immediate postoperative phase, particularly during rest. Previous studies have shown that intraoperative PAIs of corticosteroids can lead to reduced peripheral inflammation, likely due to modulation of COX-2 expression and leukocyte infiltration.^[29,30]^

In contrast, systemic intravenous (SDIV) administration leads to widespread distribution of the steroid throughout the body, allowing it to exert central and peripheral effects. This systemic exposure effectively blunts the hypothalamic–pituitary–adrenal axis response, thereby reducing systemic inflammation and providing robust antiemetic properties by acting on the chemoreceptor trigger zone and nucleus tractus solitarius. The antiemetic efficacy of intravenous dexamethasone has been well-established, as it modulates serotonergic, and neurokinin pathways involved in emesis control.^[31]^ However, because the drug is not localized to the surgical site, analgesic effects may be less pronounced in the joint space compared to PAI. This study has several limitations that should be acknowledged. First, preoperative diagnoses of the included TKA patients were not consistently reported across studies, limiting the ability to assess how underlying pathology (e.g., osteoarthritis vs rheumatoid arthritis) may have influenced pain or recovery patterns. Second, variability in patient demographics, surgical techniques, and rehabilitation protocols may have introduced unmeasured confounding. Third, the concurrent use of LIA, particularly in the PAI group, makes it difficult to isolate the specific effect of steroid administration. Finally, heterogeneity in steroid type, dose, and timing across studies, despite efforts to standardize inclusion, could also have influenced outcomes.

## 5. Conclusion

In clinical practice, intravenous steroids may be considered for patients at higher risk of PONV, while periarticular steroids may be preferred for enhancing localized pain control in the early postoperative period.

## Author contributions

**Conceptualization:** Sang Gyu Kwak, Young Woo Seo, Jae Bum Kwon, Won Kee Choi.

**Data curation:** Sang Gyu Kwak, Won Kee Choi.

**Formal analysis:** Sang Gyu Kwak, Jae Bum Kwon, Won Kee Choi.

**Investigation:** Young Woo Seo, Won Kee Choi.

**Methodology:** Sang Gyu Kwak, Won Kee Choi.

**Supervision:** Young Woo Seo.

**Validation:** Won Kee Choi.

**Visualization:** Won Kee Choi.

**Writing – original draft:** Sang Gyu Kwak, Jae Bum Kwon, Won Kee Choi.

**Writing – review & editing:** Jae Bum Kwon.
